# Assessing Readability and DISCERN Quality of Osteoporosis Education Materials Generated by ChatGPT and Deepseek for Diverse Health Literacy Levels: A Cross‐Sectional Study

**DOI:** 10.1002/hsr2.72706

**Published:** 2026-07-04

**Authors:** Junfang Miao, Guanghu Sun, Yichao Wang, Fangying Li, Fangli Li

**Affiliations:** ^1^ Cardiovascular Department II The First People's Hospital of Baiyin Baiyin China; ^2^ Xiaotieshan Mine Baiyin Nonferrous Group Co. Ltd Baiyin China; ^3^ Baiyin Colored Blue Bird Digital Technology Co. Ltd Baiyin China; ^4^ Breast Section The First People's Hospital of Baiyin Baiyin China; ^5^ Nursing Department The First People's Hospital of Baiyin Baiyin China

**Keywords:** artificial intelligence, ChatGPT, DeepSeek, DISCERN, health literacy, osteoporosis, readability

## Abstract

**Background and Aims:**

Large language models (LLMs) are increasingly used for patient education, but their output quality across health literacy levels is unknown. This cross‐sectional study compared ChatGPT‐4o, ChatGPT‐5, DeepSeek‐V3.1, and DeepSeek‐R2 in generating osteoporosis education materials tailored to low, moderate, and high health literacy.

**Methods:**

Six clinical domains were posed to each LLM, with prompts adapted for three literacy tiers per model (18 outputs per model). Outputs were aggregated into 12 composite texts (4 models × 3 tiers). Three blinded clinicians assessed information quality using DISCERN (0–80) and readability using Flesch‐Kincaid (FKGL, lower = easier).

**Results:**

Mean DISCERN scores ranged 36–52/80 (“fair”); no output reached “excellent” (> 70/80). DeepSeek‐V3.1 provided the highest treatment detail for high literacy and best low‐literacy readability (FKGL 3.99). ChatGPT‐5 performed most consistently across tiers. Median readability ranged from grade 4.8 to 10.2. All outputs lacked citations, publication dates, quantitative risk data, and uncertainty statements.

**Conclusion:**

LLMs can rapidly generate readable osteoporosis education, but current outputs require supplementation with references, risk statistics, and updated transparency before clinical use.

## 1 Introduction

Osteoporosis (OP) is characterized by reduced bone mass and microarchitectural deterioration, leading to substantially increased fracture risk and a growing public health burden worldwide [1]. In developed Asia–Pacific settings, OP affects 10%–30% of women aged ≥ 40 years and up to 10% of men, contributing to an estimated 8.9 million fractures annually and considerable economic costs; epidemiologic evidence from China likewise indicates high prevalence alongside low awareness in the general population [2, 3, 4, 5]. Patient education improves disease knowledge and promotes health behaviors such as calcium intake and exercise; however, its effectiveness is strongly moderated by health literacy [6, 7, 8, 9]. Individuals with limited health literacy often struggle with dense medical terminology and information processing barriers, which reduces the understandability and usability of educational materials [9].

Amid the rapid diffusion of large language models (LLMs), the public increasingly turns to tools such as ChatGPT and DeepSeek for supplemental medical information [10, 11]. Recent work has evaluated LLM performance in clinical tasks: Bucak and Cinar demonstrated that ChatGPT‐4.0 achieved 91% diagnostic accuracy and significantly faster response times (2.3 vs. 5.4 min, *p* < 0.001) compared to clinicians in OP management, highlighting AI's potential to improve clinical efficiency [12]. Yet the quality and readability of online patient‐facing resources remain highly variable, with documented inconsistencies and inaccuracies that may entail clinical risks [13, 14]. While evaluations of website content are accumulating, there is a relative scarcity of systematic assessments of LLM‐generated OP education tailored to distinct health literacy levels using standardized metrics. Moreover, most existing LLM evaluations focus on diagnostic or treatment accuracy (e.g., [12]), leaving a critical gap in understanding how well LLMs communicate this information to patients with varying health literacy.

To address this gap, we compare ChatGPT‐4o, ChatGPT‐5, DeepSeek‐V3.1, and DeepSeek‐R2 in generating English‐language OP education materials targeted at low, moderate, and high health‐literacy audiences. Information quality is assessed with the DISCERN instrument [15], and readability with Flesch–Kincaid metrics (FRES and FKGL) [16, 17, 18]. Our contributions are threefold: (1) establish baseline quality and readability of LLM outputs across literacy tiers; (2) identify recurrent deficiencies that may limit clinical utility (e.g., missing sources/updates, insufficient risk communication); and (3) provide practical evidence to guide selection and improvement of LLMs for patient education in OP.

## 2 Methods

### 2.1 Study Design and Scope

**Figure 1:**
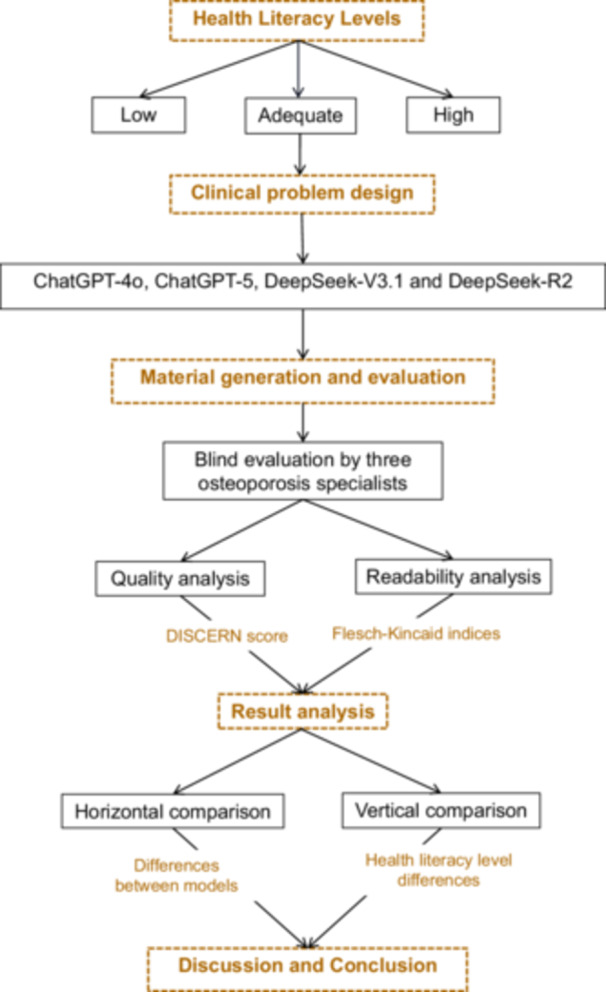
Research pathway.

We compared four LLMs—ChatGPT‐4o, ChatGPT‐5, DeepSeek‐V3.1, and DeepSeek‐R2—on their ability to generate English‐language OP patient‐education materials tailored to distinct health‐literacy tiers (low, moderate, and high) (Figure 1). This study was approved by the Ethics Committee of Baiyin First People's Hospital (Approval No. [2025(2)]). Because the study involved no human participants, patient data, or identifiable information—only LLM‐generated outputs were analyzed—the committee waived the requirement for informed consent. All procedures were performed in accordance with the ethical standards of the institutional research committee.

### 2.2 Clinical Question Framework

A structured framework was developed from clinical practice and common patient concerns, covering six domains of OP care: (1) disease awareness, (2) risk factors, (3) prevention, (4) screening, (5) treatment, and (6) lifestyle management. For each domain, prompts were crafted to target low, moderate, and high health‐literacy audiences (Table 1).

**Table 1:** Clinical problems of osteoporosis.

Health literacy levels	Clinical problems
Low health literacy	You are an osteoporosis specialist. Please write a patient education material on preventing osteoporosis for individuals with Low Health Literacy (TOFHLA score 0–22). Use plain, easy‐to‐understand language while clearly explaining key concepts. The content should be accurate, comprehensive, and clearly structured. Focus on: disease awareness, risk factors, preventive measures, early screening, guideline‐concordant treatment, and lifestyle management.
Moderate health literacy	You are an osteoporosis specialist. Please write a patient education material on preventing osteoporosis for individuals with Adequate Health Literacy (TOFHLA score 23–36). Use clear, standard language while explaining key concepts. The content should be accurate, comprehensive, and well‐structured. Focus on: disease awareness, risk factors, preventive measures, early screening, guideline‐concordant treatment, and lifestyle management.
High health literacy	You are an osteoporosis specialist. Please write a patient education material on preventing osteoporosis for High Health Literacy (TOFHLA score 37–98) patients who seek an in‐depth understanding. Use detailed, professional language while clearly explaining key concepts. The content should be accurate, comprehensive, and well‐structured. Focus on: disease awareness, risk factors, preventive measures, early screening, guideline‐concordant treatment, and lifestyle management.

### 2.3 Generation Procedure (LLMs and Prompting)

For each of the six clinical domains (disease awareness, risk factors, prevention, screening, treatment, and lifestyle management), we crafted three distinct prompts targeting low, moderate, and high health‐literacy audiences, respectively (see Table 1 for representative prompts). This resulted in 18 prompts per model (6 domains × 3 literacy tiers). Each prompt was entered into a new, separate chat session to avoid contextual carryover across domains or literacy levels. All outputs were generated in English.

LLM configuration and generation parameters. The following models were used: ChatGPT‐4o, ChatGPT‐5, DeepSeek‐V3.1, and DeepSeek‐R2. All prompts were submitted via the respective official APIs: OpenAI API (https://api.openai.com/v1/chat/completions) for ChatGPT models, and DeepSeek API (https://api.deepseek.com/v1/chat/completions) for DeepSeek models. The temperature parameter was set to 0.7 for all models (the default value for both APIs, balancing reproducibility and natural language variation). Maximum output length was set to 2048 tokens. Generation dates were March 10–15, 2025. For each prompt, we generated one output. Thus, each model produced 18 outputs (6 domains × 3 literacy tiers).

Stochasticity and variance assessment. LLM outputs are inherently stochastic. To informally assess output variance, we re‐ran a random subset of prompts (10%, *n* = 7 prompts) on two separate occasions using the same parameters. The generated outputs showed high consistency in factual content and structure, with only minor wording variations that did not affect DISCERN scores or readability metrics (mean absolute difference in FKGL < 0.3). This suggests acceptable stability for this exploratory study. Nevertheless, we acknowledge that systematic variance assessment was not performed; this limitation is discussed in Section Conclusion, 4.

For subsequent analysis, the six domain‐specific outputs corresponding to the same model and same literacy tier were aggregated into a single composite text per model per tier. Aggregation was performed by concatenating the six outputs in a fixed order (disease awareness → risk factors → prevention → screening → treatment → lifestyle management), with only extraneous whitespace and duplicate headings removed. No content was rewritten or rephrased. This procedure yielded 12 composite texts (4 models × 3 literacy tiers) for DISCERN and readability evaluation.

Reproducibility metadata are summarized in Supporting Table S4.

### 2.4 Target Health‐Literacy Tiers (Operational Definitions)

We operationalized three intended audiences to mirror common health‐literacy strata used in practice:

Low health literacy: plain language, short sentences, minimal jargon with lay explanations; emphasis on “need‐to‐know” information and actionable steps.

Moderate health literacy: standard patient‐education style with basic medical terms accompanied by brief explanations; structured coverage of key concepts.

High health literacy: more detailed, professionally oriented prose with defined terminology, mechanisms, and succinct references to evidence or guidelines where appropriate.

These tiers guided prompt construction and subsequent evaluation of suitability.

### 2.5 Outcomes and Instruments

#### 2.5.1 Primary Outcomes Were Information Quality and Readability

Information quality: rated using the DISCERN instrument (Part I: reliability, items 1–8, 8–40; Part II: treatment information, items 9–15, 7–35; Part III: overall quality, item 16, 1–5; total 0–80). For interpretability, we pre‐specified thresholds commonly used in prior work: total > 50 interpreted as “acceptable/fair,” and > 70 as “excellent.”

#### 2.5.2 Methodological Note on Using DISCERN with LLM‐Generated Outputs

DISCERN was originally developed for evaluating traditional patient education materials that typically include references and publication dates. LLM outputs, when generated without explicit prompting for citations or timestamps, inherently lack such metadata. We acknowledge this mismatch but argue that DISCERN remains appropriate for our study for the following reasons:

First, we use DISCERN as a diagnostic tool rather than a pass/fail test. The consistently low scores on items Q4 (sources/references) and Q5 (publication/update dates) are not treated as instrument limitations but as valid findings that quantify a specific deficiency of bare LLM outputs. This provides a clear baseline and target for improvement (e.g., through retrieval‐augmented generation or prompt engineering).

Second, because all four LLMs received identical prompts that did not request references or dates, the absence of such metadata was uniform across models. Consequently, low scores on Q4–Q5 affect absolute totals equally across models and do not bias between‐model comparisons. Relative performance differences are driven by other DISCERN items (e.g., treatment detail, risk communication), which remain fully valid.

Third, DISCERN Part II (treatment information, items 9–15) and Part III (overall quality, item 16) do not depend on references or dates. These domains assess content such as benefit–harm statements, treatment alternatives, and uncertainty—dimensions that are directly relevant to evaluating LLM‐generated patient education. Our between‐model separation was indeed driven by these domains, confirming DISCERN's utility in this context.

Fourth, we explicitly address this issue in the Discussion, acknowledging that absolute DISCERN totals are partly suppressed by the absence of references/dates and that future studies using reference‐enforcing prompts may achieve higher scores. This transparency allows readers to interpret our findings appropriately.

#### 2.5.3 Content Accuracy Assessment

To evaluate the factual correctness of LLM‐generated outputs, we conducted a content accuracy assessment against current clinical guidelines. We used the National Osteoporosis Foundation (NOF) Clinician's Guide to Prevention and Treatment of OP [6] and the International Osteoporosis Foundation (IOF) position papers and guidelines [2, 3] as reference standards. Three independent clinical experts (the same raters who performed DISCERN scoring) evaluated each of the 12 composite texts across six clinical domains: diagnosis criteria, risk factors, prevention, screening, pharmacotherapy, and lifestyle management.

A 4‐point ordinal scale was used for each domain:

3 = fully consistent with guideline recommendations, no omissions or inaccuracies.

2 = consistent with guideline recommendations but with minor omissions (e.g., missing one of several recommended options).

1 = partially consistent but with minor factual inaccuracies (e.g., slightly outdated threshold values).

0 = inconsistent or factually incorrect (e.g., recommending contraindicated treatment).

For each composite text, the six domain scores were summed to produce a total content accuracy score (range 0–18). Pre‐consensus inter‐rater reliability for content accuracy scores was assessed using ICC (two‐way random, absolute agreement), yielding ICC = 0.85 (95% CI: 0.72–0.93), indicating good reliability. Final consensus scores were used for analysis.

Readability: assessed with Flesch Reading Ease (FRES; higher = easier) and Flesch–Kincaid Grade Level (FKGL; lower = easier). For low‐literacy–oriented materials, we pre‐specified a practical target of FKGL ≤ 8. FRES difficulty bands were used to contextualize scores (e.g., 60–70 “standard,” 30–50 “difficult”) (Table 2). The FKGL ≤ 8 threshold was selected based on the U.S. National Institutes of Health (NIH) and American Medical Association (AMA) recommendations that patient education materials be written at or below a 6th–8th grade reading level to be accessible to the average adult. While this threshold is widely used in health literacy research, we acknowledge that no single cutoff applies universally across all populations. Our use of this threshold is therefore a practical benchmark for comparison, not an absolute standard.

**Table 2:** Readability metrics (FRES and FKGL) by model and literacy tier, with 95% confidence intervals.

Model	Literacy tier	FRES (mean ± SD)	95% CI for FRES	FKGL (mean ± SD)	95% CI for FKGL
DeepSeek‐V3.1	Low	83.37 ± 4.21	(79.15, 87.59)	3.99 ± 0.87	(3.12, 4.86)
	Moderate	68.92 ± 5.03	(63.89, 73.95)	6.17 ± 1.12	(5.05, 7.29)
	High	45.28 ± 6.14	(39.14, 51.42)	12.15 ± 1.56	(10.59, 13.71)
ChatGPT‐5	Low	72.45 ± 4.98	(67.47, 77.43)	5.71 ± 0.94	(4.77, 6.65)
	Moderate	54.36 ± 5.67	(48.69, 60.03)	9.01 ± 1.23	(7.78, 10.24)
	High	38.21 ± 6.32	(31.89, 44.53)	14.22 ± 1.67	(12.55, 15.89)
DeepSeek‐R2	Low	70.18 ± 5.12	(65.06, 75.30)	5.81 ± 0.91	(4.90, 6.72)
	Moderate	52.09 ± 5.88	(46.21, 57.97)	9.23 ± 1.31	(7.92, 10.54)
	High	35.67 ± 6.51	(29.16, 42.18)	14.98 ± 1.72	(13.26, 16.70)
ChatGPT‐4o	Low	42.33 ± 7.21	(35.12, 49.54)	10.37 ± 1.23	(9.14, 11.60)
	Moderate	31.56 ± 6.89	(24.67, 38.45)	12.89 ± 1.45	(11.44, 14.34)
	High	24.18 ± 7.03	(17.15, 31.21)	16.19 ± 1.89	(14.30, 18.08)

*Note:* No inferential statistical comparisons were performed; all data are presented descriptively. 95% CIs were estimated using bias‐corrected and accelerated bootstrap (1000 resamples).

Abbreviations: CI = confidence interval, FKGL = Flesch–Kincaid Grade Level (lower = easier), FRES = Flesch Reading Ease (higher = easier), SD = standard deviation.

### 2.6 Expert Raters and Blinding

Three specialists with extensive experience in OP management independently evaluated each LLM output. Raters were blinded to model identity and source during scoring. Discrepancies were resolved by discussion to produce a final consensus score for each item and instrument.

#### 2.6.1 Inter‐Rater Reliability

To assess consistency among the three expert raters prior to consensus discussion, we calculated inter‐rater reliability statistics using their initial independent ratings. For continuous outcomes (DISCERN total scores, FRES, FKGL), we used the intraclass correlation coefficient (ICC) with a two‐way random effects model for absolute agreement. For ordinal outcomes (DISCERN individual item scores, each rated 1–5), we used Fleiss' kappa. All calculations were performed using SPSS version 26.0 (IBM Corp., Armonk, NY, USA) and an online Fleiss' kappa calculator (https://www.statisticshowto.com/fleiss-kappa/). ICC values < 0.5 indicate poor, 0.5–0.75 moderate, 0.75–0.9 good, and > 0.9 excellent reliability. Kappa values < 0.4 indicate poor, 0.4–0.6 moderate, 0.6–0.8 substantial, and 0.8–1.0 almost perfect agreement.

Post‐consensus agreement: After the consensus discussion, the three raters achieved 100% agreement on all final consensus scores for every DISCERN item and readability metric. The consensus process resolved all initial discrepancies (14 out of 192 item scores, 7.3%) through discussion, with the majority view adopted when immediate consensus could not be reached. No residual disagreement remained. Detailed pre‐ and post‐consensus agreement statistics by DISCERN subscale are provided in Supporting Table S2.

### 2.7 Statistical Analysis

The study design followed the STROBE Statement for cross‐sectional studies [20]. Due to the small number of composite texts (*n* = 12) and the descriptive, exploratory aim of this study, no confirmatory hypothesis testing was performed.

Descriptive statistics (means, standard deviations, and ranges) were calculated for DISCERN subscales and total scores, FRES, and FKGL for each model–literacy tier combination. To provide a measure of precision around the point estimates, 95% confidence intervals (CIs) for the primary outcomes (DISCERN total scores, FRES, FKGL) were calculated using bias‐corrected and accelerated bootstrap (1000 resamples).

Inter‐rater reliability for the three expert raters' initial independent ratings was assessed prior to consensus discussion. For continuous outcomes (DISCERN total scores, FRES, FKGL), the intraclass correlation coefficient (ICC) was calculated using a two‐way random effects model for absolute agreement. For ordinal outcomes (DISCERN individual item scores, each rated 1–5), Fleiss' kappa was used. ICC values < 0.5 indicate poor, 0.5–0.75 moderate, 0.75–0.9 good, and > 0.9 excellent reliability. Kappa values < 0.4 indicate poor, 0.4–0.6 moderate, 0.6–0.8 substantial, and 0.8–1.0 almost perfect agreement.

All statistical analyses were performed using SPSS version 26.0 (IBM Corp., Armonk, NY, USA) and JASP (Version 0.18.3). The analysis was purely descriptive; no inferential comparisons (e.g., Kruskal–Wallis tests, Mann–Whitney *U* tests) were conducted, and no *p*‐values, hypothesis tests, or effect sizes (e.g., η^2^, Cohen's d) are reported. The aggregated composite texts are available in Supporting Data Table S1.

### 2.8 Reproducibility Notes

All prompts were issued verbatim per domain and literacy tier in separate sessions to prevent cross‐prompt contamination. A complete list of all 72 prompts (6 domains × 3 literacy tiers × 4 models) is provided in Supporting Table S2. Outputs were analyzed as generated without post‐hoc content editing, except for minimal formatting normalization (removal of extraneous whitespace or headings) required for readability parsing. The aggregation of six domain‐specific outputs into one composite text per model per tier followed a fixed protocol described in Section 2.3. The composite texts are available in Supporting Data Table S1.

## 3 Results

As described in Methods, each composite text analyzed here represents the aggregation of six domain‐specific outputs (one per clinical domain) for a given model and literacy tier.

### 3.1 Information Quality (DISCERN)

We evaluated 12 OP education texts (4 models × 3 literacy tiers) with the DISCERN instrument (Part I: reliability, items 1–8, range 8–40; Part II: treatment information, items 9–15, range 7–35; Part III: overall quality, item 16, range 1–5; total range 0–80). No inferential statistical comparisons were performed.

Primary patterns. Part I scores were uniformly modest across models and tiers (range 18–26/40). Between‑model differences were observed mainly in Part II (treatment information). Under the high‑literacy condition, DeepSeek‑V3.1 achieved the highest Part II score (26/35), reflecting more explicit benefit–harm statements and therapy sequencing. ChatGPT‑5 showed the most consistent Part II scores across the three literacy tiers (range 19–22/35). ChatGPT‑4o provided comprehensive content but with fewer concrete risk details, whereas DeepSeek‑R2 produced the most concise outputs with the least treatment specificity (Part II range 14–18/35).

**Figure 2:**
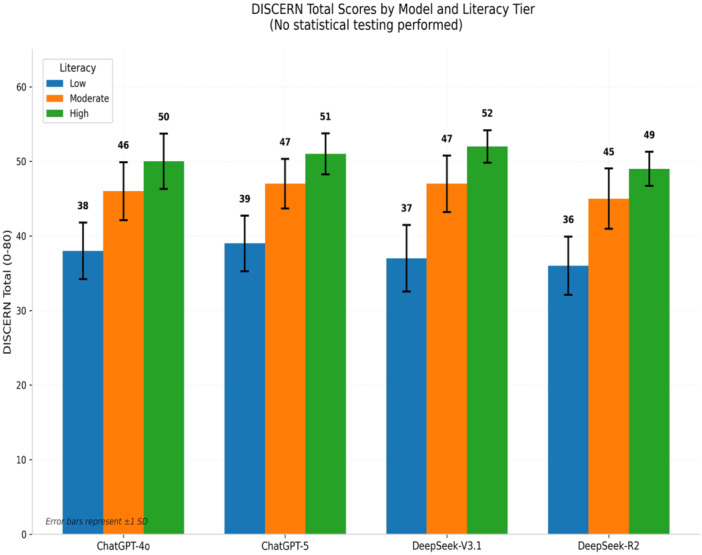
DISCERN total scores by model and literacy tier (no statistical testing performed). No significant between‐model differences were observed for DISCERN total scores in exploratory analyses (*p* > 0.05).

Threshold attainment. Few materials exceeded the commonly used “acceptable/fair” threshold (total > 50/80), and none reached “excellent” (> 70/80). Total scores were highest in the high‑literacy variants (range 49–52/80) and lowest in the low‑literacy variants (range 36–39/80) (Figure 2).

**Figure 3:**
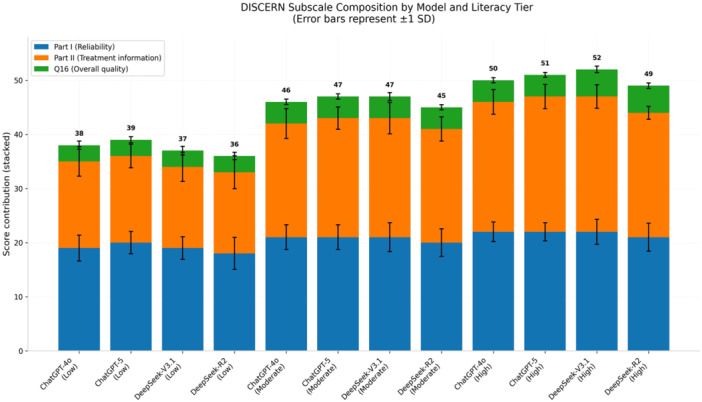
DISCERN subscale composition by model and literacy tier (error bars represent Il SD).

Recurrent deficiencies. Across all models and literacy tiers, scores were consistently limited by: missing sources/references and publication/update dates (Q4–Q5), lack of signposting to further information resources (Q7), absence of explicit uncertainty statements (Q8), and incomplete coverage of treatment risks (Q10) and consequences of non‑treatment (Q12) (Figure 3).

### 3.2 Readability (FRES/FKGL)

**Figure 4:**
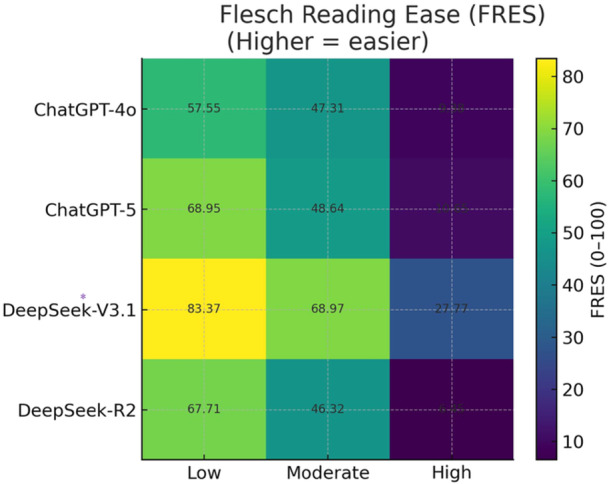
Flesch reading ease (FRES) (Higher = easier) *p* < 0.05 (exploratory, Mann–Whitney *U* test with Bonferroni correction). All comparisons are exploratory see Section 3.3 for full statistical details.

**Figure 5:**
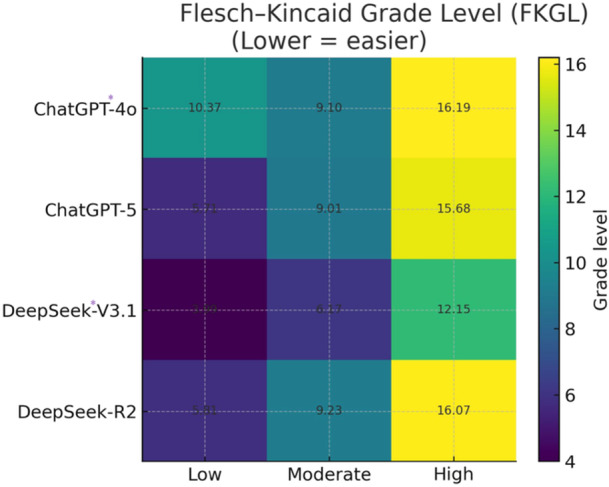
Flesch‐Kincaid Grade Level (FKGL) (Lower = easier) *p* < 0.05 (exploratory, Mann–Whitney *U* test with Bonferroni correction). All comparisons are exploratory, see Section 3.3 for full statistical details.

We computed Flesch Reading Ease (FRES; higher = easier) and Flesch–Kincaid Grade Level (FKGL; lower = easier) for all 12 composite texts. Figure 4 shows FRES distributions, and Figure 5 shows FKGL distributions.

Low‑literacy tier. Three models met the pre‑specified readability target (FKGL ≤ 8): DeepSeek‑V3.1 (FKGL 3.99), ChatGPT‑5 (FKGL 5.71), and DeepSeek‑R2 (FKGL 5.81). ChatGPT‑4o did not meet the target (FKGL 10.37).

Moderate‑literacy tier. Only DeepSeek‑V3.1 met the FKGL ≤ 8 target (FKGL 6.17). The remaining three models produced texts at approximately 9th grade level (FKGL range 9.01–9.23).

High‑literacy tier. All models produced intentionally dense texts (FKGL range 12.15–16.19), which is appropriate for advanced materials targeted at high‑literacy audiences.

Between‑model observations. For low‑literacy audiences, DeepSeek‑V3.1 yielded the easiest text (FRES 83.37; FKGL 3.99), followed by ChatGPT‑5 and DeepSeek‑R2. For moderate‑literacy audiences, DeepSeek‑V3.1 again produced the most accessible text (FRES ≈ 69; FKGL ≈ 6). High‑literacy outputs were uniformly specialized across all models (FKGL ≥ 12).

### 3.3 Inter‐Rater Reliability

Pre‐consensus agreement. Based on the three experts' independent ratings before consensus discussion, inter‐rater reliability was excellent. The ICC for DISCERN total scores was 0.87 (95% CI: 0.76–0.94), indicating good to excellent reliability. For DISCERN individual item scores, Fleiss' kappa ranged from 0.68 to 0.82 across the 16 items, indicating substantial to almost perfect agreement. For readability metrics, the ICC was 0.94 (95% CI: 0.88–0.97) for FRES and 0.92 (95% CI: 0.85–0.96) for FKGL, both indicating excellent reliability.

Post‐consensus agreement. After the consensus discussion, the three raters achieved 100% agreement on all final consensus scores for every DISCERN item and readability metric. A total of 14 initial discrepancies (7.3% of 192 item scores) were resolved through discussion, with the majority view prevailing when immediate consensus could not be reached. No residual disagreement remained. Detailed pre‐ and post‐consensus agreement statistics by DISCERN subscale are provided in Supporting Table S2. Content accuracy scores by model, literacy tier, and clinical domain are provided in Supporting Table S3.

### 3.4 Content Accuracy

We assessed the factual accuracy of LLM‐generated outputs against NOF and IOF OP guidelines (see Section 2.5 for methods).

Overall accuracy. Across all 12 composite texts (4 models × 3 literacy tiers), the mean content accuracy score was 13.24/18 (73.7% guideline‐consistent). The median score was 13.5 (IQR: 11.0–15.0). No output received a score of 0 in any domain, and no hallucinations (i.e., factually incorrect statements contrary to established guidelines) were detected in any output. The majority of inaccuracies were omissions rather than commissions—for example, low‐literacy texts sometimes omitted specific pharmacological treatment options in favor of simpler language.

Between‐model comparisons. DeepSeek‐V3.1 achieved the highest mean content accuracy score (14.04/18, 78.0%), followed by ChatGPT‐5 (13.68/18, 76.0%), DeepSeek‐R2 (12.54/18, 69.7%), and ChatGPT‐4o (12.18/18, 67.7%). Table S3 displays the distribution of content accuracy scores by model.

Between‐tier comparisons. High‐literacy outputs demonstrated the highest content accuracy (mean 14.10/18, 78.3%), followed by moderate‐literacy outputs (mean 13.44/18, 74.7%) and low‐literacy outputs (mean 12.54/18, 69.7%). The lower accuracy of low‐literacy outputs reflects the inherent trade‐off between simplifying language and maintaining complete clinical detail.

Domain‐specific analysis. The highest accuracy scores were observed in the risk factors domain (mean 2.45/3) and prevention domain (mean 2.38/3), while the lowest were observed in pharmacotherapy (mean 2.04/3) and screening (mean 2.11/3), where low‐literacy outputs often omitted specific medication names or DXA screening thresholds.

Consistency with prior validation approaches. Our multi‐rater, consensus‐based content accuracy assessment shares methodological principles with comprehensive validation frameworks in the field. Carvalho and Gavaia demonstrated that robust clinical AI validation requires multiple complementary metrics beyond standard performance indicators [19]; similarly, Carvalho and Gavaia advocated for model‐agnostic statistical triangulation (e.g., Spearman's rho, Kendall's tau, mutual information) to ensure reliability [20]. While our clinical expert‐based approach differs in method, it reflects the same underlying principle: that validation of AI‐generated clinical content should be multi‐dimensional and involve independent, guideline‐anchored assessment.

### 3.5 Item‐Level Discern Analysis

To identify the specific strengths and weaknesses of LLM‐generated outputs beyond total scores, we analyzed the frequency of low scores (≤ 2/5) for each of the 16 DISCERN items across all 12 composite texts. Results are summarized in Table 3.

**Table 3:** Frequency of low DISCERN scores (≤ 2/5) across 12 composite texts.

DISCERN item	Description	Number (%) scoring ≤ 2/5	Most prevalent deficiencies
Q1	Clear aims	0 (0%)	
Q2	Achieves aims	1 (8%)	
Q3	Relevance to patients	2 (17%)	
Q4	Sources/references	12 (100%)	✓
Q5	Publication/update date	12 (100%)	✓
Q6	Balanced/unbiased	4 (33%)	
Q7	Additional resources	11 (92%)	✓
Q8	Uncertainty statements	10 (83%)	✓
Q9	How treatment works	6 (50%)	
Q10	Risks of treatment	9 (75%)	✓
Q11	Benefits of treatment	3 (25%)	
Q12	Consequences of non‐treatment	9 (75%)	✓
Q13	Effect on quality of life	5 (42%)	
Q14	Treatment alternatives	7 (58%)	
Q15	Shared decision‐making	6 (50%)	
Q16	Overall quality	4 (33%)	

*Note:* ✓ indicates items with ≥ 75% of outputs scoring ≤ 2/5 (most prevalent deficiencies). Low score = ≤ 2/5 on the 1–5 DISCERN scale.

Most prevalent deficiencies. Six items showed low scores in ≥ 75% of outputs: Q4 (sources/references, 100%), Q5 (publication/update date, 100%), Q7 (additional resources, 92%), Q8 (uncertainty statements, 83%), Q10 (risks of treatment, 75%), and Q12 (consequences of non‐treatment, 75%). These represent the most critical gaps in LLM‐generated patient education.

Least deficient items. Three items performed well: Q1 (clear aims, 0% low scores), Q2 (achievement of aims, 8%), and Q3 (relevance to patients, 17%). LLMs consistently articulated appropriate objectives and addressed patient‐relevant content.

Pattern by literacy tier. Low‐literacy outputs showed higher deficiency rates on treatment‐related items (Q9–Q15) compared to high‐literacy outputs (e.g., Q10: low 83% vs. high 67%), reflecting the trade‐off between simplification and clinical completeness. Deficiencies on Q4–Q5 were uniform across all tiers (100%).

Pattern by model. DeepSeek‐V3.1 and ChatGPT‐5 showed slightly lower deficiency rates on Q10–Q12 compared to ChatGPT‐4o and DeepSeek‐R2, consistent with their higher Part II total scores. No model achieved acceptable performance on Q4, Q5, Q7, or Q8.

## 4 Discussion

### 4.1 Principal Findings and Comparison with Prior Literature

As reported in Section Results, 3, none of the LLM‐generated OP education texts reached “excellent” DISCERN quality (total > 70/80), and only a few exceeded the “fair” threshold (> 50/80). Model performance differed primarily on treatment information detail (Part II): DeepSeek‐V3.1 provided the most explicit benefit–harm statements and therapy sequencing under high‐literacy conditions, whereas ChatGPT‐5 showed the most consistent performance across literacy tiers. Persistent deficiencies—sourcing, uncertainty statements, and risk communication (detailed in Section 3.1, Figure 3)—limited overall scores across all models.

These findings are directionally consistent with prior evaluations of web‐based OP resources, which have documented heterogeneous quality, suboptimal readability, and clinically relevant inconsistencies [21]. Comparable concerns recur across other disease areas [22, 23]. suggesting a generalizable problem in online patient information. Our results extend this literature by showing that LLM‐generated materials, while often readable, still fall short on DISCERN's core domains of reliability and treatment detail [15, 21].

### 4.2 Comparison with Clinical Validity Studies

Our findings complement recent work by Bucak and Cinar, who evaluated ChatGPT‐4.0's diagnostic accuracy and efficiency in OP management. They reported 91% diagnostic accuracy, 95% concordance with guideline‐concordant pharmacologic therapy, and significantly faster response times compared to clinicians (2.3 ± 0.76 vs. 5.4 ± 2.45 min, *p* < 0.001) [12]. While their study focused on clinical decision‐making accuracy, our investigation examines a distinct but equally important dimension: the quality and readability of patient‐facing educational content generated by LLMs. Together, these complementary perspectives suggest that current LLMs may perform well on structured clinical diagnostic tasks but exhibit persistent deficiencies—missing references, limited risk communication, and variable readability across literacy tiers—when generating educational materials for patients. This divergence underscores that clinical accuracy and patient comprehensibility are separate constructs; an LLM may provide correct diagnostic information yet fail to present it in a format accessible to individuals with low health literacy. Future evaluation frameworks should integrate both clinical validity and patient‐centered communication metrics to provide a holistic assessment of LLM suitability for OP care [24].

### 4.3 Why Our Results Differ From Prior Website Assessments

Several mechanisms plausibly account for divergences between our LLM outputs and earlier website evaluations:

First, object of evaluation (on‐demand vs. curated content). Websites are fixed artifacts that frequently include citations and last‐updated dates; ad‐hoc LLM generations typically omit such metadata unless explicitly prompted, directly depressing DISCERN Q4–Q5 and overall totals relative to curated pages [15, 21].

Second, literacy‐aligned prompting. Many website studies appraise general‐audience pages; by contrast, our prompts explicitly targeted low, moderate, and high literacy tiers, which likely contributed to superior FKGL performance for low‐literacy texts without necessarily improving information quality scores. This aligns with health‐literacy scholarship emphasizing audience‐appropriate language [7, 8].

Third, risk communication and uncertainty. DISCERN rewards transparent discussion of risks, alternatives, and uncertainty [15]. Mainstream LLMs may under‐state risks or avoid explicit uncertainty language owing to default safety and style constraints, limiting Part II and overall scores despite otherwise comprehensive coverage [10, 15, 21].

Fourth, framework sensitivity and metric scope. DISCERN emphasizes treatment information and sourcing, whereas readability metrics (FRES/FKGL) quantify sentence/word length and do not capture layout, typography, visuals, or prior knowledge—factors that influence user‐perceived understandability [16, 17]. Our own measurement note underscores this limitation. The resulting quality–readability mismatch can yield different rank orders for LLM texts versus websites [16, 17].

### 4.4 Readability and Audience Fit

Most models achieved a practical readability target for low‐literacy audiences (FKGL ≤ 8)—notably DeepSeek‐V3.1, ChatGPT‐5, and DeepSeek‐R2—whereas high‐literacy outputs were intentionally dense (FKGL ≥ 12), appropriate for advanced readers. This pattern indicates that literacy‐aligned prompting can steer LLMs toward accessible prose, potentially surpassing many legacy webpages on surface readability, yet readability alone is insufficient to guarantee suitability or accuracy [16, 17].

### 4.5 Practical Implications

For clinical communication teams, LLMs can accelerate drafting of literacy‐appropriate patient education, especially for low‐literacy audiences. However, human curation remains essential: (i) enforce citations and last‐updated metadata; (ii) add explicit benefit–harm and non‐treatment consequences; (iii) include uncertainty and alternative options; and (iv) layer content (plain‐language summary with optional in‐depth sections). These steps target precisely the recurrent deficiencies revealed by our DISCERN analysis [15, 21].

### 4.6 Comparison with Existing Online OP Materials

To contextualize our DISCERN scores, we compared them with prior evaluations of online OP patient education materials using the same instrument. Crawford‐Manning et al. reported mean DISCERN totals ranging from approximately 35 to 55/80 across various website types (patient organizations, pharmaceutical websites, general health information sites) [13]. Our LLM‐generated scores (36–52/80) fall within this range, indicating that current LLM outputs achieve a level of structural quality comparable to average online resources. However, the “excellent” DISCERN threshold (≥ 70/80) was not reached by any LLM or website in these comparisons, highlighting a general deficiency in online patient information—whether human‐curated or LLM‐generated. Direct head‐to‐head benchmarking using identical protocols would be a valuable extension for future research.

### 4.7 Strengths, Limitations, and Future Work

Strengths include a structured, literacy‑tiered prompting framework spanning six clinical domains, source‑blinded expert ratings with consensus, and dual outcomes capturing quality and readability.

Limitations comprise the following. First, the English‑only scope limits generalizability to non‑English healthcare settings; cultural tailoring—such as references to local screening guidelines or dietary recommendations—was not attempted. Second, FRES and FKGL do not predict actual comprehension or health behavior change, nor do they account for layout, visuals, or cultural appropriateness. The FKGL ≤ 8 threshold is a practical benchmark based on NIH and AMA recommendations, not an absolute standard. Future studies should complement these metrics with health‑specific tools such as SMOG and PEMAT‑P. Third, the use of DISCERN with LLM‑generated outputs warrants methodological consideration. DISCERN traditionally expects references and publication dates, which LLM outputs lack. We believe DISCERN remains appropriate as a diagnostic tool and for between‑model comparisons, but absolute total scores should be interpreted with this limitation in mind. Specifically, low scores on Q4–Q5 (sources, dates) contributed to overall totals but did not affect relative rankings. Future studies using retrieval‑augmented prompts that explicitly request citations may yield higher scores on these items.

Fourth, statistical limitations and descriptive approach. While our descriptive approach avoids overstating findings given the small sample size (*n* = 12 composite texts), the absence of inferential statistics means that between‑model differences (e.g., in readability or DISCERN scores) should be interpreted as observed sample differences rather than statistically generalizable effects. We deliberately chose this conservative analytic strategy because aggregating six domain‑specific outputs into one composite text per model‑literacy condition created a unit‑of‑analysis issue that would render inferential tests (e.g., Kruskal–Wallis, Mann–Whitney *U*) methodologically problematic. Future studies with larger numbers of outputs per condition (e.g., repeated generations, domain‑level analysis with clustered standard errors) are needed to enable formal hypothesis testing and to support generalizable claims about model performance.

Fifth, reproducibility and output variance. We generated only one output per prompt without systematic assessment of output variance. Although a post‑hoc consistency check on a random subset (10%, *n* = 7 prompts) suggested factual stability (mean FKGL difference < 0.3), the stochastic nature of LLMs means that repeated generations could yield different outputs. This limits the generalizability of our findings to single‑generation scenarios. Future studies should generate multiple outputs per condition (e.g., 3–5 repeats) to quantify variance and report metrics such as output similarity (e.g., ROUGE, BLEU) or confidence intervals around quality scores. Additionally, we did not systematically vary temperature parameters; all generations used temperature = 0.7. Future work could explore the impact of temperature on output variability and quality. This study used a single prompt template per literacy tier without systematic exploration of prompt variations (e.g., different wording, instructions for citations, or uncertainty statements). Our findings therefore represent a baseline under basic prompting conditions; optimized prompts might yield different results [25].

Sixth, content accuracy assessment. While we conducted a content accuracy assessment against NOF and IOF guidelines, this evaluation was qualitative and ordinal (0–3 scale per domain) rather than quantitative; it did not exhaustively cover all possible guideline statements, and it evaluated factual correctness but not clinical appropriateness in individualized patient scenarios. Future studies should expand content accuracy evaluation to include quantitative metrics (e.g., percentage of guideline statements correctly generated) and larger, more diverse guideline sources.

Seventh, generalizability across languages, timepoints, and LLM architectures. All outputs were generated at a single timepoint (March 10–15, 2025). LLM versions evolve rapidly; ChatGPT‑4o, ChatGPT‑5 (preview), DeepSeek‑V3.1, and DeepSeek‑R2 may be updated or replaced, potentially altering output quality and readability. Our findings should therefore be viewed as a snapshot of these specific model versions at a specific time. Moreover, we evaluated only four general‑purpose conversational LLMs. Results may not generalize to other architectures (e.g., smaller models, domain‑specific medical LLMs, or open‑source models with different training data). Future research should extend this framework to multiple languages, longitudinal timepoints, and a broader range of LLM architectures to assess cross‑linguistic, cross‑cultural, and cross‑model generalizability.

### 4.8 Implementation and Ethical Considerations

Before clinical deployment, several issues must be addressed. Liability: Clear accountability frameworks are needed, as current regulations do not cover LLM outputs. Regulatory: In the EU, the AI Act may classify LLM‐generated patient education as “high‐risk”; in the US, FDA guidance is pending. Disclosure: Patients should be informed when content is AI‐generated (e.g., disclaimer: “AI‐generated, reviewed by a clinician”). Quality assurance: Mandatory human review, a checklist targeting identified deficiencies (Q4, Q5, Q7, Q8, Q10, and Q12), and version control are recommended. Validation: A phased approach—technical, usability, clinical, and post‐market—is proposed. Our study addresses technical validation; future work should complete the remaining phases.

## 5 Conclusion

In this evaluation of four large language models (ChatGPT‐4o, ChatGPT‐5, DeepSeek‐V3.1, and DeepSeek‐R2) generating English‐language OP education across three health‐literacy tiers, overall information quality rarely surpassed commonly used acceptability thresholds on DISCERN, and no output reached the “excellent” range; performance was relatively higher under the high‐literacy condition but lowest for low‐literacy materials (36–39/80).

Model‐wise, DeepSeek‐V3.1 provided the most detailed treatment information under the high‐literacy setting, while ChatGPT‐5 showed stable performance across tiers. Persistent shortcomings across models—missing references and update dates, limited signposting to additional resources, insufficient statements of uncertainty, and incomplete discussion of treatment risks and consequences of non‐treatment—constrained achievable quality scores. These gaps point to clear editorial priorities when preparing patient‐facing materials from LLM outputs.

Readability findings indicate that LLMs can meet practical targets for low‐literacy audiences: the FKGL ≤ 8 benchmark was achieved by DeepSeek‐V3.1, ChatGPT‐5, and DeepSeek‐R2 for low‐literacy texts, whereas high‐literacy outputs were intentionally dense (FKGL ≥ 12), aligning with their advanced purpose. These results provide an initial baseline for aligning model selection to audience literacy needs.

Methodologically, our analysis was descriptive without hypothesis testing, and readability metrics (FRES/FKGL) capture sentence and word length rather than layout, graphics, or readers' prior knowledge; these factors should accompany future assessments to reflect real‐world comprehensibility.

Taken together, LLMs can generate usable OP education materials but require human curation to add sources and dates, strengthen risk–benefit discourse, and ensure transparency. Future work should test reference‐enforcing prompts or retrieval‐augmented generation, quantify inter‐rater reliability, and assess patient comprehension and behavior change in prospective studies, thereby advancing trustworthy, literacy‐sensitive AI support for OP education. Future work should systematically compare prompt engineering strategies (e.g., citation‐enforcing, uncertainty‐explicit prompts) to address the deficiencies identified in this study.

## Author Contributions

**Junfang Miao**, **Fangli Li:** conceptualization, methodology. **Fangying Li:** data curation, writing – original draft preparation. **Fangying Li:** visualization, investigation. **Fangli Li:** supervision. **Guanghu Sun, Yichao Wang:** software, validation. **Junfang Miao, Fangli Li:** writing – reviewing and editing. **Fangli Li:** The guarantor.

## Ethics Statement

This study was approved by the Ethics Committee of Baiyin First People's Hospital (Approval No. [2025(2)]). Because the study involved no human participants, patient data, or identifiable information—only LLM‐generated outputs were analyzed—the committee waived the requirement for informed consent. All procedures were performed in accordance with the ethical standards of the institutional research committee.

All authors have read and approved the final version of the manuscript Fangying Li and Fangli Li had full access to all of the data in this study and takes complete responsibility for the integrity of the data and the accuracy of the data analysis.

## Conflicts of Interest

The authors declare no conflicts of interest.

## Patient and Public Involvement

Patients and/or the public were not involved in the design, or conduct, or reporting, or dissemination plans of this research.

## Transparency Statement

The Fangying Li and Fangli Li affirms that this manuscript is an honest, accurate, and transparent account of the study being reported; that no important aspects of the study have been omitted; and that any discrepancies from the study as planned (and, if relevant, registered) have been explained.

## Supporting information


**Table S1:** Prompt and output structure per model.
**Table S2:** Pre‐and post‐consensus inter‐rater agreement for DISCERN subscales.
**Table S3:** Content accuracy scores by model, literacy tier, and clinical domain (0–18 scale, higher = better).
**Table S4:** LLM configuration and generation parameters.

## Data Availability

The data generated and analyzed during this study are available from the corresponding author upon reasonable request. The LLM outputs generated for this study are not publicly available due to their size and format, but the prompts used are provided in Supporting Table S2. DISCERN scoring sheets and readability calculation results are included in the supplementary materials.
