# Supplementary material for: Assessing Readability and DISCERN Quality of Osteoporosis Education Materials Generated by ChatGPT and Deepseek for Diverse Health Literacy Levels: A Cross‐Sectional Study
Source: Health Sci Rep. 2026 Jul 4;9(7):e72706. doi: 10.1002/hsr2.72706 (PMC13332862; doi:10.1002/hsr2.72706)
Supplement: Supplementary file 1 — Table S1: Prompt and output structure per model. Table S2: Pre‐and post‐consensus inter‐rater agreement for DISCERN subscales. Table S3: Content accuracy scores by model, literacy tier, and clinical domain (0–18 scale, higher = better). Table S4: LLM configuration and generation parameters. [file HSR2-9-e72706-s001.docx]

### Supplementary Table S1. Prompt and output structure per model

| **Component** | **Count per model** | **Explanation** |
| --- | --- | --- |
| Clinical domains | 6 | Disease awareness, risk factors, prevention, screening, treatment, lifestyle management |
| Health literacy tiers | 3 | Low, moderate, high |
| **Prompts per model** | **18** | 6 domains × 3 tiers |
| **Outputs generated per model** | **18** | One per prompt |
| **Composite texts per model** | **3** | 6 domain outputs aggregated by literacy tier |
| **Total composite texts analyzed** | **12** | 4 models × 3 literacy tiers |

Note on aggregation: For each model and literacy tier, the six domain-specific outputs were concatenated in a fixed order without content modification. The resulting composite text was used for DISCERN and readability scoring.

**Supplementary Table S2. Pre-and post-consensus inter-rater agreement for DISCERN subscales**

| **DISCERN subscale/metric** | **Pre-consensus statistic(ICC/Fleiss’kappa)** | **95%Cl** | **Post-consensus agreement(%)** |
| --- | --- | --- | --- |
| Part I (Reliability,items 1-8) | ICC▒=▒0.81 | 0.68-0.89 | 100% |
| Part Il (Treatment information,items 9-15) | ICC▒=▒0.85 | 0.73-0.92 | 100% |
| Part Ⅲ(Overallquality,item 16) | Fleiss’kappa =0.76 | 0.61-0.91 | 100% |
| DISCERN total score | ICC▒=▒0.87 | 0.76-0.94 | 100% |
| FRES | ICC▒=▒0.94 | 0.88-0.97 | 100% |
| FKGL | ICC▒=▒0.92 | 0.85-0.96 | 100% |

Note: Post-consensus agreement represents the proportion of final consensus scores on which all three raters agreed after discussion. For all metrics, 100% agreement was achieved.

Supplementary Table S3. Content accuracy scores by model, literacy tier, and clinical domain (0-18 scale, higher = better)

| **Model** | Literacy tier | **Diagnosis** | Risk factors | **Prevention** | **Screening** | **Pharmacotherapy** | **Lifestyle** | Total(max 18) | %guideline-consistent |
| --- | --- | --- | --- | --- | --- | --- | --- | --- | --- |
| DeepSeek-V3.1 | Low | 2.33 | 2.67 | 2.50 | 2.17 | 2.00 | 2.50 | 14.17 | 78.7% |
| DeepSeek-V3.1 | Moderate | 2.50 | 2.83 | 2.67 | 2.33 | 2.17 | 2.50 | 15.00 | 83.3% |
| DeepSeek-V3.1 | High | 2.67 | 3.00 | 2.83 | 2.50 | 2.33 | 2.67 | 16.00 | 88.9% |
| ChatGPT-5 | Low | 2.17 | 2.50 | 2.33 | 2.00 | 1.83 | 2.33 | 13.16 | 73.1% |
| ChatGPT-5 | Moderate | 2.33 | 2.67 | 2.50 | 2.17 | 2.00 | 2.50 | 14.17 | 78.7% |
| ChatGPT-5 | High | 2.50 | 2.83 | 2.67 | 2.33 | 2.17 | 2.67 | 15.17 | 84.3% |
| DeepSeek-R2 | Low | 1.83 | 2.33 | 2.17 | 1.83 | 1.67 | 2.17 | 12.00 | 66.7% |
| DeepSeek-R2 | Moderate | 2.00 | 2.50 | 2.33 | 2.00 | 1.83 | 2.33 | 13.00 | 72.2% |
| DeepSeek-R2 | High | 2.17 | 2.67 | 2.50 | 2.17 | 2.00 | 2.50 | 14.00 | 77.8% |
| ChatGPT-4o | Low | 1.67 | 2.17 | 2.00 | 1.67 | 1.50 | 2.00 | 11.01 | 61.2% |
| ChatGPT-4o | Moderate | 2.00 | 2.33 | 2.17 | 1.83 | 1.67 | 2.17 | 12.17 | 67.6% |
| ChatGPT-4o | High | 2.17 | 2.50 | 2.33 | 2.00 | 1.83 | 2.33 | 13.16 | 73.1% |

*Note: Scores are consensus scores from three expert raters. Pre-consensus ICC▒=▒0.85 (95% Cl:0.72-0.93). Domain scores are means of the three domain-specific items within each category.*

| Supplementary Table S4.LLM configuration and generation parameters  Parameter | **ChatGPT-4o** | **ChatGPT-5** | **DeepSeek-V3.1** | **DeepSeek-R2** |
| --- | --- | --- | --- | --- |
| **Exact** **version** | 2024-05-13 | 2025-01-25 preview | 2025-02-18 | 2025-03-01 |
| **APlendpoint** | <https://api.openai.com/v1/chat/completions> | <https://api.openai.com/v1/chat/cmpletions> | <https://api.deepseek.com/v1/chat/completio>  ns | <https://api.deepseek.com/v1/chat/completio>  ns |
| **Temperature** | 0.7 | 0.7 | 0.7 | 0.7 |
| **Max** **tokens** | 2048 | 2048 | 2048 | 2048 |
| **Top-p** | 1.0 | 1.0 | 1.0 | 1.0 |
| **Frequency** **penalty** | 0 | 0 | 0 | 0 |
| **Presence** **penalty** | 0 | 0 | 0 | 0 |
| **Generation** **date** **range** | March 10-15,2025 | March 10-15,2025 | March 10-15,2025 | March 10-15,2025 |
| **Number** **of** **outputs** **generated** **per** **model** | 18 | 18 | 18 | 18 |
| **Number** **of** **composite** **texts** **analyzed** **per**  **model** | 3 | 3 | 3 | 3 |

Note: All prompts were submitted in separate chat sessions to avoid contextual carryover. Nosystem prompts or prior conversation history were used. The temperature of 0.7 was chosen asthe default value for both APIs, balancing reproducibility with natural language variation.
